# Supplementary material for: Imaging of Acute Abdominopelvic Pain in Pregnancy and Puerperium—Part II: Non-Obstetric Complications
Source: Diagnostics (Basel). 2023 Sep 11;13(18):2909. doi: 10.3390/diagnostics13182909 (PMC10528125; doi:10.3390/diagnostics13182909)
Supplement: Supplementary file 1 [file diagnostics-13-02909-s001.zip › diagnostics-2562035-supplementary.pdf]

Complication	US	MRI	CT
Acute appenditicis	Dilated (>6–7 mm in diameter), aperistaltic, noncompressible, and thick-walled (>2 mm) tubular structure arising from the cecum.<br>Periappendiceal hyperechoic inflamed fat or hypoechoic fluid.	Distended appendix with hyperintense lumen (T2-weighted images).<br>Periappendiceal fluid and inflammation (T2-weighted images FS).<br>High signal on DWI and restricted diffusion within appendiceal wall.	Alternative if MRI is not available.<br>Enlarged and wall thickened appendix with stranding of the adjacent fat.
Small bowel obstruction	Low accuracy	Dilated and fluid-filled loops with levels and transition point (T2-weighted images). Submucosal wall oedema (T2-weighted images FS). Transition point may not be seen due to thick sections. Mesenteric oedema and ascites are also observed.	Alternative if MRI is not available.
Inflammatory bowel disease	Low accuracy	Bowel wall circumferentially thickening greater than 3 mm, mucosal ulcerations, submucosal oedema (hyperintense on T2 weighted images), bowel lumen narrowing with upstream dilatation. Coomb sign. Creeping fat.	Alternative if MRI is not available.
HELLP<br>• Hepatic infarction<br><br>• Hepatic hematoma<br><br>• Hepatic rupture	Non specific findings.	Imaging modality of choice in pregnancy.<br><br>Subcapsular area of hypersignal (on T2 weighted images), with decreased or absent enhancement after intravenous administration of a Gadolinium chelate (T1 weighted images).<br><br>Heterogeneous space-occupying lesion, that compress the adjacent parenchyma.<br>Variable appearance according to the age of the bleeding.<br><br>Not indicated.	First diagnostic method in puerperium.<br><br>Large and markedly hypoattenuating, peripheral area with persisting foci of enhancement (mottled appearance). Lack of compression on the surrounding parenchyma.<br><br>Same MRI findings.<br>Active extravasation of contrast medium can be detected (active bleeding).<br><br>Direct loss of liver capsule integrity associated with liver hematoma and haemoperitoneum.<br>Active extravasation of contrast medium can be detected (active bleeding).
Acute cholecystitis<br><br><br>Acute pancreatitis	Dilated gallbladder (short axis diameter > 3 cm), with wall thickening (> 3 mm) and pericholecystic fluid. Dependent sludge and/or gallstones can also be detected.<br>Positive US Murphy's sign.<br><br>Although it has low diagnostic value for pancreatitis, it can reliably detect gallstones and biliary dilatation.	Should be performed after inconclusive US.<br>PPV of up to 100% in diagnosing AC.<br>Hydropic distention, gallbladder wall thickening with edematous stratification, and pericholecystic inflammatory changes (T2 weighted images FS). Stones are depicted as filling defects on heavily T2-weighted thin-cut sequences.<br><br>Enlarged oedematous pancreas.<br>Peripancreatic fat stranding and free fluid.<br>Heterogeneity of normal T1 hyperintense parenchyma suggests necrosis. Look for gallstone disease (most common cause).	Alternative if MRI is not available.
Urolithiasis	First imaging test despite its<br>poor sensitivity (11-24%).	Accurate in differentiating physiological from pathological urinary dilation. Small stones best seen on heavily T2-weighted	Alternative if MRI is not available and in unresolved cases, to depict obstructing urolithiasis.

Pyelonephritis	Doppler techniques can be useful in detecting urolithiasis.	coronal sequence as filling defect with upstream dilatation.<br>Renal enlargement or perinephric fat stranding/ oedema. Foci of nefritis are depicted on DWI as wedge shaped areas of high signal intensity.	
Uterine Leyomioma red degeneration	Accurate.<br>Heterogeneous or hyperechoic lesions and later anechoic components, reflecting cystic changes. Absent flow on Color-doppler.	Helpful diagnostic adjunct when leiomyomas are located deep in the pelvis or in the posterior myometrium.<br>Fibroid is T2 intermediate or increased signal, and T1 high signal	Not indicated.
Ovarian Torsion	Unilateral enlarged ovary (> 5 cm) with or without a pre-existing mass.<br>Multiple peripherally displaced follicles with echogenic stroma , vascular pedicle twisting (whirpool sign). Free pelvic fluid.	Should be performed after inconclusive US. Enlarged ovary due to venous congestion and oedema (high T2 signal) Peripheralised T2 hyperintense follicles. T1 hyperintense haemorrhagic foci. Vascular pedicle twisting (a beak-like protrusion adjacent to the enlarged ovary). Look for underlying lesion (e.g. dermoid).	Not indicated.
Ovarian vein thrombosis	Less sensitive than CT and MRI.<br>The thrombosed vein is enlarged with an intra-luminal echogenic mass. On the colour flow Doppler, there is reduced or no flow within the lumen of the vessel but there may be increased flow surrounding the vein due to the perivascular inflammatory reaction	Problem solving tool in pregnancy. The thrombus can exhibit variable signal intensity, depending on the age of luminal blood products. Unenhanced MR venography performed with TOF technique can depict a flow void in the vessel or the vessel may be completely absent.	Imaging modality of choice in puerperium.<br>Presence of a filling defect within the ovarian vein on contrast enhanced CT.<br>Enlargement of the vein and enhancement of the vessel wall may be observed.

Table S1. Key imaging features of the different non obstetrical causes of acute abdominopelvic pain during pregnancy.
